# Spontaneous Subcutaneous Emphysema and Pneumomediastinum Associated With Influenza B Virus in a Young Male Adult: A Case Report

**DOI:** 10.7759/cureus.13077

**Published:** 2021-02-02

**Authors:** Wafa Y Alnofal, Majedah R Alshadely, Mohammad A Khatib

**Affiliations:** 1 College of Medicine, King Saud Bin Abdulaziz University for Health Sciences, Riyadh, SAU; 2 Internal Medicine, Prince Sultan Military Medical City, Riyadh, SAU

**Keywords:** spontaneous, subcutaneous emphysema, pneumomediastinum, influenza b, saudi arabia

## Abstract

In this report, we present a case of influenza B-associated spontaneous pneumomediastinum (SPM) and subcutaneous emphysema (SE) in a young male patient. Although there are several causes responsible for this condition, it is considered extremely rare to occur spontaneously with no prior trauma or predisposing factors, especially in older age groups.

Rare cases of SPM and SE, as described in several case reports worldwide, have been reported to have a benign course for the most part. Nevertheless, implementing unified management guidelines with clear protocols is highly recommended to prevent the potential unfavorable complications associated with these conditions.

## Introduction

Spontaneous pneumomediastinum (SPM) and subcutaneous emphysema (SE) are uncommon conditions, in which spontaneous refers to the leakage of air from the lung cavity to other cavities including the subcutaneous tissue, without causing any traumas. Usually, it is self-limiting or may require supportive management only, unless it is caused by a serious underlying pathology of the lungs or associated with critical conditions such as pneumothorax and secondary bacterial pneumonia [1].

In 15 countries within the Asia-pacific region, influenza B has been identified in 0-92% of influenza cases. Rather than subtypes, influenza B is separated into two lineages that could co-circulate: B/Yamagata and B/Victoria. Vaccine strains used for each of these two lineages are antigenically different and only offer minimal cross-protection. In children aged between 1-10 years, influenza B is associated with higher rates of attacks, but serious complications that may necessitate hospitalization are mainly detected in older age groups, immunocompromised patients, and individuals with other comorbidities [2].

The incubation period is usually around two days from the onset of the infection, after which symptom presentation occurs, including fever, dry cough, fatigue, sore throat, and headache. It could also lead to severe complications such as secondary bacterial infections and worsening of existing medical conditions [3].

In this report, we present a case of SPM with SE, which was most likely caused or triggered by the influenza B virus, in a previously healthy male; the patient was managed successfully with supportive care alone.

## Case presentation

A 23-year-old military trainee male with a significant history of cigarette smoking presented to the emergency room (ER) with clinical symptoms of fatigue, weakness, oliguria, nausea, and vomiting for two days. On review of systems, the patient reported mild dysphagia with throat and neck pain, in addition to a history of subjective fever before presenting to the ER. On physical examination, crepitations were felt on palpation of the neck.

Initial laboratory workup showed increased urea and creatinine levels (14.5 mmol/L and 501 μmol/L, respectively), as well as slightly decreased sodium and corrected calcium levels (132 mmol/L and 1.92 mmol/L, respectively). Significantly elevated creatinine kinase levels (998 U/L) and mildly raised C-reactive protein (21 mg/L) were also evident on workup done upon admission, consistent with acute kidney injury secondary to dehydration and rhabdomyolysis due to strenuous physical exercise as part of the patient's military training. Complete blood count with differentials revealed elevated white blood cells (11.7 x 10^9^/L) with high absolute neutrophils (9 x 10^9^/L). The liver function test was unremarkable except for a raised aspartate transaminase level (78 U/L). 

**Figure 1:**
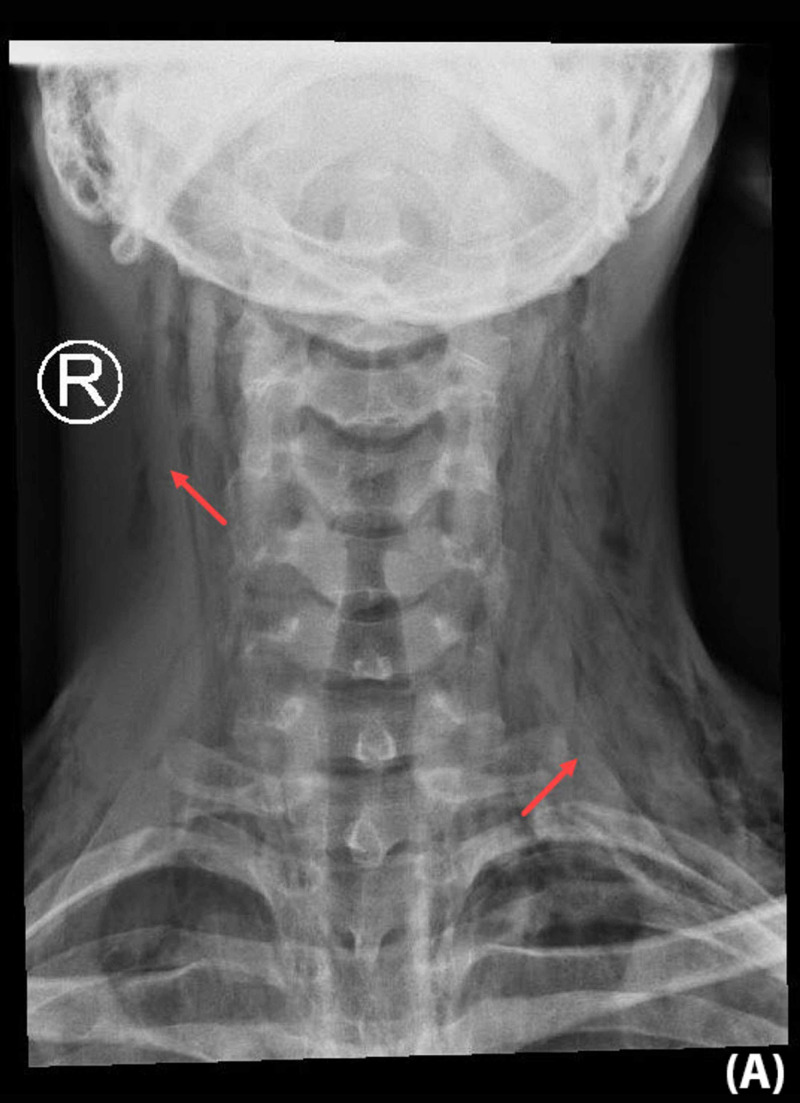
Anteroposterior neck X-ray demonstrated subcutaneous emphysema in the soft tissue of the neck and upper chest wall

**Figure 2:**
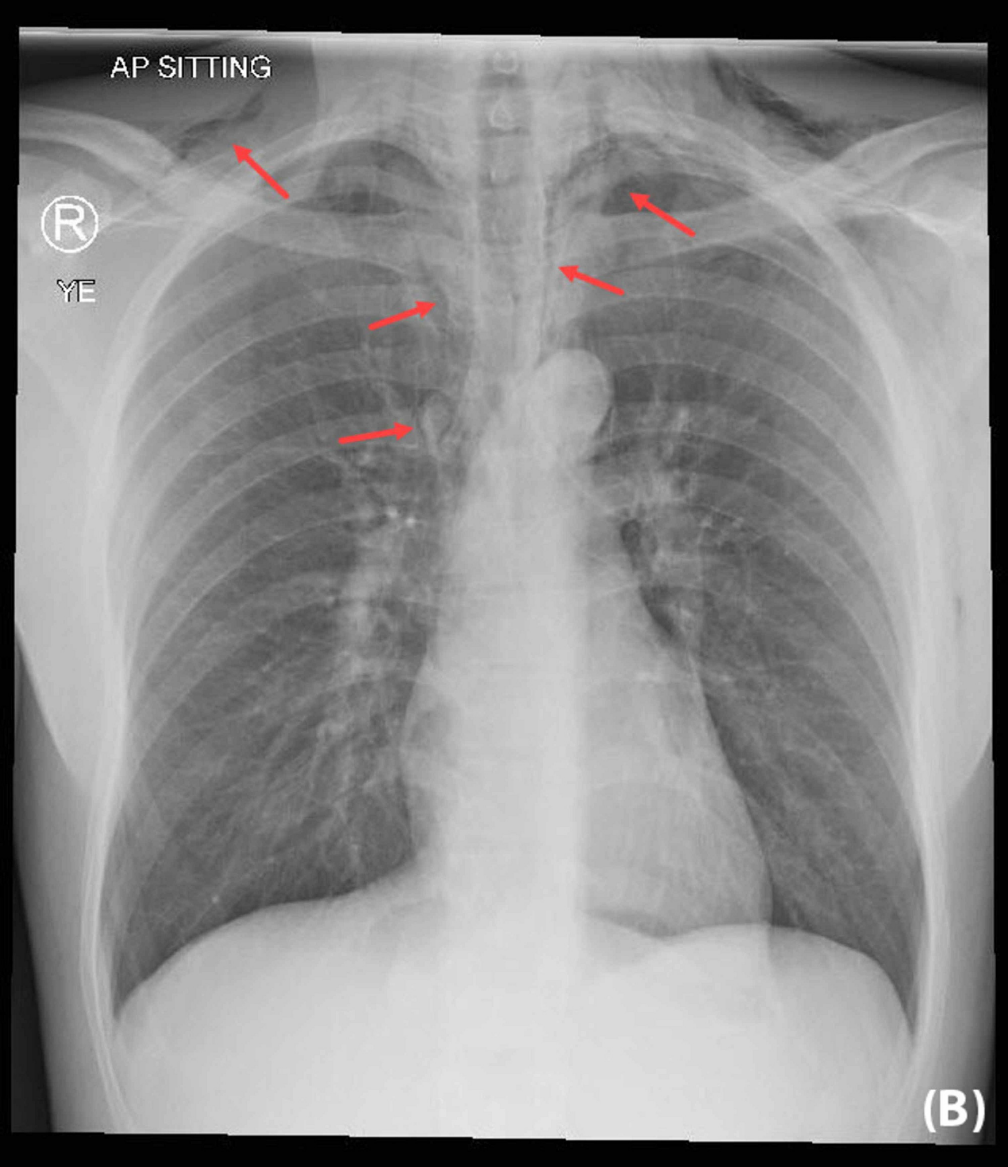
Chest X-ray showed bilateral subcutaneous emphysema within the upper chest wall Small pneumomediastinum was also noted with no focal lung lesion appreciated

Urgent X-rays of the neck and chest revealed bilateral SE in the lower part of the neck and upper chest with minimal pneumomediastinum. No pneumothorax or pleural effusion was noted (Figure 1, Figure 2).

**Figure 3:**
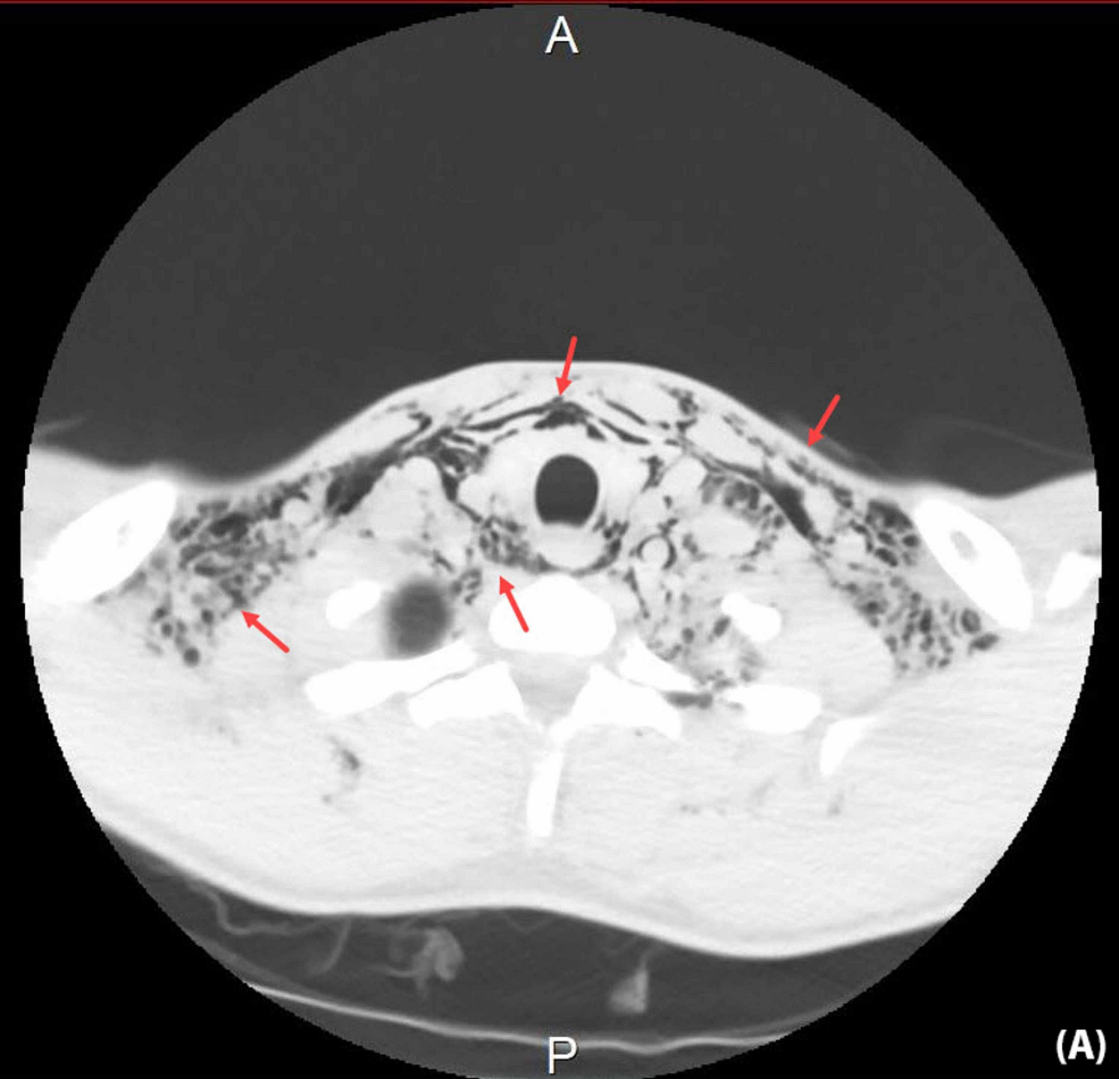
Non-contrast axial head and neck CT scan The scan revealed subcutaneous emphysema involving the retropharyngeal space, surrounding the esophagus and extending between the neck fascia down into the upper chest, left axilla, supraclavicular regions as well as the mediastinum CT: computed tomography

**Figure 4:**
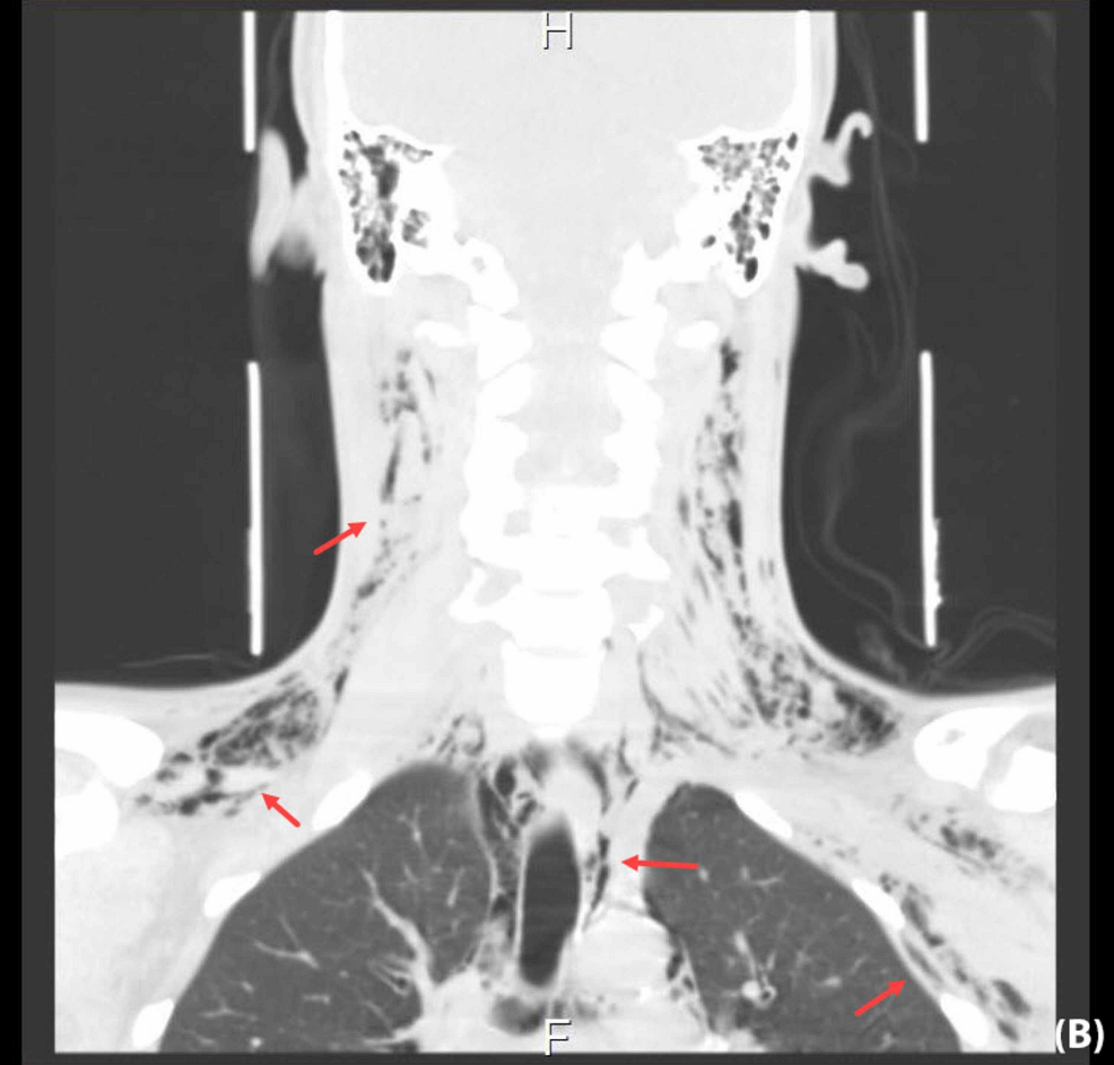
Non-contrast coronal head and neck CT scan The scan revealed subcutaneous emphysema involving the retropharyngeal space, surrounding the esophagus and extending between the neck fascia down into the upper chest, left axilla, supraclavicular regions as well as the mediastinum CT: computed tomography

Non-contrast-enhanced CT scan of the head and neck showed similar findings to the X-rays but with a more detailed view of the soft-tissue extension (Figure 3, Figure 4).

**Figure 5:**
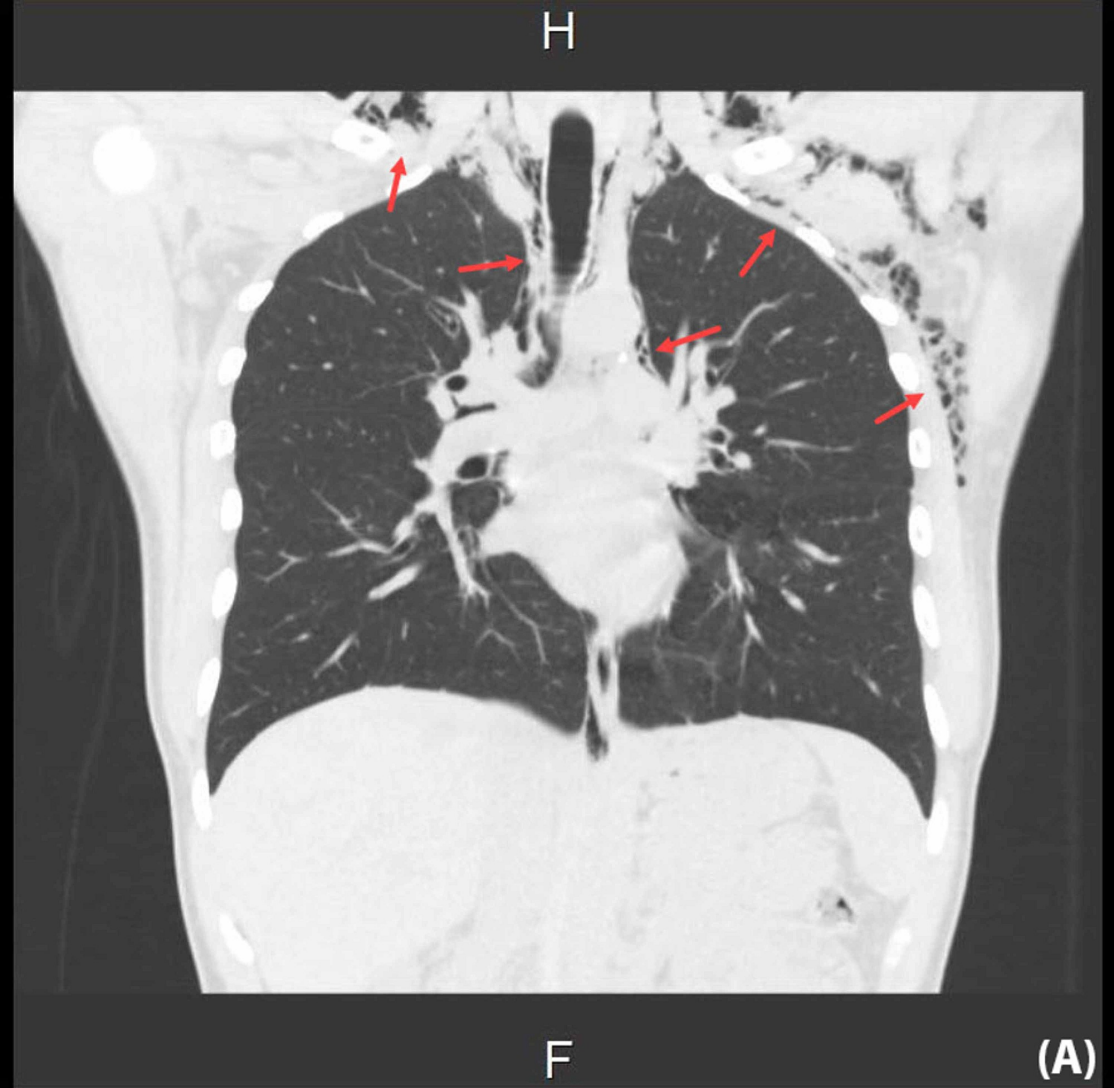
Non-contrast coronal CT chest The CT displayed bilateral extensive subcutaneous emphysema extending into the anterior and posterior upper part of the chest, and supraclavicular and left axillary regions in addition to pneumomediastinum CT: computed tomography

**Figure 6:**
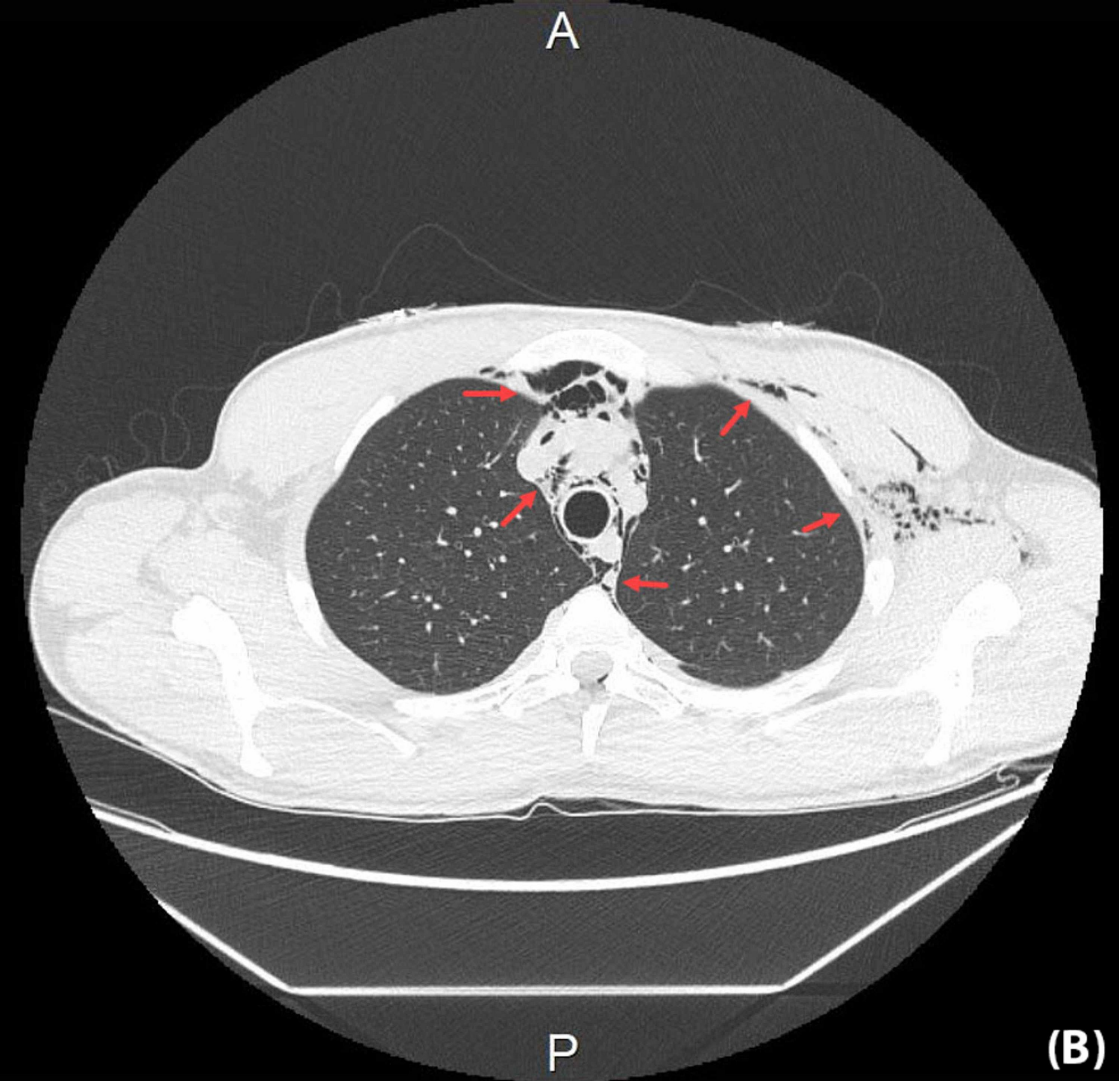
Non-contrast axial CT chest The CT displayed bilateral extensive subcutaneous emphysema extending into the anterior and posterior upper part of the chest, and supraclavicular and left axillary regions in addition to pneumomediastinum CT: computed tomography

Chest CT scan without contrast was also performed to rule out causes of the SE and SPM; no detectable etiology was found (Figure 5, Figure 6).

Perinuclear anti-neutrophil cytoplasmic antibodies, cytoplasmic anti-neutrophil cytoplasmic antibodies, and anti-glomerular basement membrane antibodies were negative. Serological tests for hepatitis B, C, and HIV antibodies were also negative. Bacterial cultures from blood, urine, and sputum samples were all negative. Lastly, polymerase chain reaction done on obtained nasopharyngeal swab came back positive for influenza B virus.

The patient was managed conservatively with oxygen supplementation through a nasal cannula and the acute kidney injury responded well to IV fluid hydration, in addition to the resolution of rhabdomyolysis confirmed with normalized creatinine kinase levels. Repeated complete blood count with differential during hospitalization and post hydration revealed leukopenia (2.8 x 10^9^/L) with low lymphocyte count and absolute neutrophils (1.2 x 10^9^/L and 1.4 x 10^9^/L, respectively). The patient was started on oseltamivir 75 mg BID for five days. PIP/TAZ was briefly started owing to suspicion of hospital-acquired infection due to fever (>38 °C) and slightly elevated procalcitonin (0.15 ng/mL) on the next day; however, it was stopped within 24 hours due to transaminitis, which self-resolved.

After a few days of supportive therapy along with oseltamivir, significant improvement in the patient’s symptoms was noted. The examination also revealed the resolution of crepitation on palpation. Repeated complete blood count with differential showed normalized levels of all parameters except absolute neutrophils, which were slightly lower than normal (1.4 x 10^9^/L).

On the fifth day of hospitalization, marked resolution of SE and SPM was confirmed by a chest X-ray done on discharge. Unfortunately, the patient missed his three-month follow-up appointment, and follow-up could not be performed.

## Discussion

SE and SPM are mostly traumatic or post-surgical, as in the case of barotrauma leading to rupture of alveoli and subsequent air leakage to the mediastinum and adjacent cervical subcutaneous cavities [1]. However, although it is rare, these conditions can occur due to non-traumatic causes including infections and abuse of illicit drugs, in addition to chronic respiratory conditions such as asthma, which result in the weakening of the bronchoalveolar walls, thereby making them fragile and prone to rupture [1].

When both conditions occur simultaneously, they are known as Hamman’s syndrome, which is associated with elevated intra-thoracic pressure due to different causes including straining as in the case of our patient, mostly due to the heavy military training, and can present with Hamman's sign, which is the sound of a click or a crunch made of a beating heart against air-filled tissue at the apex and along the left heart border [4].

Influenza virus infection can induce SPM and SE by damaging the alveolar cell wall, or by intense cough, or both [5]. Published cases from the literature have shown similar presentations and courses of management regarding several strains of influenza like H1N1 [6], H5N6 [7], and even influenza B [8]. Other than influenzas, the infectious causes of SPM and SE also include tuberculosis [9] and chronic sinusitis with recurrent aggressive coughs [10]. Non-infectious causes can be due to cocaine use with 3,4-Methyl​enedioxy​methamphetamine (MDMA) [11].

The most common presenting symptoms include, but are not limited to, chest and neck pain, dyspnea, cough, nasal voice, and dysphagia, with signs on examination including tenderness with crepitus sensation over the areas of emphysema, pulsus paradoxus, tachycardia, and Hamman's sign in cases of massive SE [6].

SE and SPM are essentially known to be benign entities with uncomplicated hospital course in most cases. However, although rarely, if the pneumomediastinum pressure is sufficient, it can lead to adverse complications such as tension pneumomediastinum and pneumothorax, which may cause cardiac function compromise and tamponade as well as respiratory distress and subsequent failure, unless proper management measures are promptly established [12,13].

As per history, investigations, and radiology, there was no detectable cause for SPM and SE in our patient, no pre-existing lung disease, or history of trauma/surgery. However, smoking, as wells as strenuous physical activities and weightlifting required in daily military training can precipitate the condition along with the aggressive cough-associated influenza B infection of the upper respiratory tract leading to the resultant extensive SPM and SE.

## Conclusions

The presented case showed the possible association of the influenza B virus with the development of SPM and SSE. The patient was successfully managed with oxygen, fluids, and antivirals without significant complications and a benign hospital course. Screening for influenza virus in patients with atypical presentations, especially during the season of influenza, followed by appropriate management of the symptoms and underlying cause can majorly affect outcomes.
